# Epicardial ventricular tachycardia ablation: safety and efficacy of access and ablation using low-iodine content

**DOI:** 10.1007/s00392-024-02378-6

**Published:** 2024-02-07

**Authors:** Julian Müller, Ivaylo Chakarov, Philipp Halbfass, Karin Nentwich, Artur Berkovitz, Kai Sonne, Sebastian Barth, Heiko Lehrmann, Thomas Deneke

**Affiliations:** 1 Clinic for Interventional Electrophysiology, Heart Centre Bad Neustadt, Von-Guttenberg-Straße 11, 97616 Bad Neustadt an Der Saale, Germany; 2 Department of Cardiology, Faculty of Medicine, University Heart Center Freiburg-Bad Krozingen, University of Freiburg, Freiburg, Germany; 3 Department of Cardiology and Angiology, Philipps-University Marburg, Marburg, Germany; 4 Department of Cardiology, Klinikum Oldenburg, European Medical School Oldenburg-Groningen, Carl Von Ossietzky University, Oldenburg, Germany

**Keywords:** Epicardial ablation, Anterior epicardial access, VT ablation, Sudden cardiac death, Mortality, Hospitalization

## Abstract

**Background:**

Epicardial ablation has become an integral part of the treatment of ventricular tachycardias (VT). This study reports the safety of epicardial access as well as the efficacy of epicardial ablation of structural heart disease in a tertiary single-center experience.

**Methods:**

Between January 2016 and February 2022, consecutive patients undergoing an epicardial access for VT ablation were included. Different puncture techniques and occurrence of epicardial access-related complications as well as the safety of ablation using non-ionic 5% dextrose in water (D5W) compared to standard 0.9% normal saline (NS) irrigation were analyzed. VT recurrence rates during a mean follow-up of 37 ± 23 months were reported.

**Results:**

In total, 197 patients undergoing a total of 239 procedures were included (59.8 ± 15.3 years, 86% males). A total of 154 patients (78%) had non-ischemic cardiomyopathies with a mean LVEF of 37 ± 14. Anterior-oriented epicardial access was aimed for in all cases and was successful in 217 (91%) of all procedures, whereas access was achieved in 19 procedures (8%) only using an inferior oriented access and in three procedures (1%) using surgical access due to severe adhesions or anatomical requirements. Overall epicardial puncture-related complications occurred in 18 (8%) of all procedures with minor pericardial bleeding in nine, pericardial tamponade in one, pneumothorax in five, pneumopericardium in one, and abdominal puncture in two cases. Presence of adhesions could be identified as the only independent predictor of epicardial access-related complications. D5W was used in 79 cases and regular 0.9% saline in 117 procedures. No differences were seen regarding acute ablation success or complications. During follow-up, 47% of all patients were free from any VTs (56% D5W vs. 40% NS; log-rank *p* = 0.747) and 92% of clinical VTs (98% D5W vs. 91% NS; log-rank *p* = 0.139).

**Conclusions:**

In this large single-centre experience, epicardial access and ablation were safe and feasible. Although long-term clinical VT recurrence rates were low, overall VT recurrences as well as mortality were high advocating for a highly experienced, interdisciplinary approach including intense management of underlying cardiac disease/heart failure. Routine usage of D5W was safe and associated with comparable short- or long-term clinical or overall VT freedom.

**Graphical abstract:**

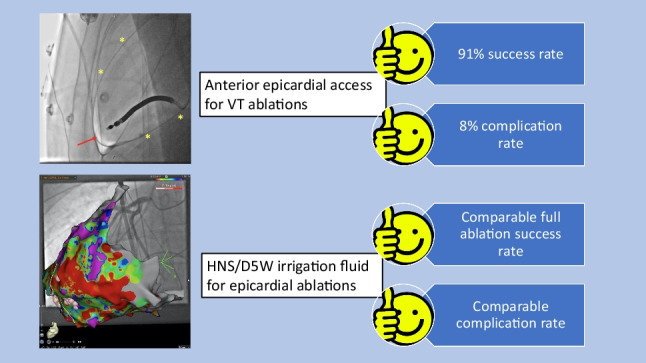

## Introduction

Ventricular tachycardia (VT) often complicates advanced stages of structural heart diseases and is an important cause of sudden cardiac death (SCD) [1]. In the past decades, catheter ablation of sustained VTs has become an important and effective treatment option to reduce VT recurrences and prolong ICD shock-free survival [2, 3]. Despite improvements in endocardial ablation techniques, imaging and electroanatomic mapping VT recurrences are still common, especially in non-ischemic patients with complex, often epicardial substrates. The first report of a percutaneous posterior subxiphoid approach to access the epicardial space was published by Sosa et al. in patients with Chagas disease [4]. Since then, the indication for gaining epicardial access has expanded for a wide range of cardiomyopathies and ablation procedures [5, 6]. Epicardial ablations may improve outcomes; however, the associated complication rates remain high including devastating complications such as pericardial tamponade, abdominal organ puncture, or inadvertent right ventricular injury requiring immediate cardiac surgery [7, 8].

Different puncture techniques are used in clinical practise. Initially, the dry subxiphoid puncture of the pericardial space has been performed aiming for the inferior pericardial space (“inferior approach”) [4] including the risk of injuries of adjacent structures such as the abdominal organs [7]. An “anterior” approach aims to puncture underneath the xiphoid towards the anterior right ventricular epicardial space limiting potential of collateral damage. This technique accesses directly the pericardium without passing through the diaphragm [9].

For patients undergoing epicardial catheter ablation of VT, current guidelines recommend irrigated tip radiofrequency (RF) ablation to optimize lesion characteristics and applicability. However, no specific composition of the irrigation fluid is proposed but may have relevant consequences on the risk profile and achievable lesion geometry. Low-ionic concentration fluids can be used to reduce energy shunting by creating a milieu of a higher impedance- lower electrical conductivity- irrigant cloud during RF delivery directing energy flow towards the myocardium. Therefore, in this study we sought to compare the safety of (1) epicardial access techniques and (2) our updated irrigation management with non-ionic 5% dextrose in water (D5W) irrigation fluid compared to normal saline (NS) for VT ablation and (3) the long-term outcome after epicardial VT ablation in a large single-center cohort.

## Methods

### Study population

**Fig. 1:**
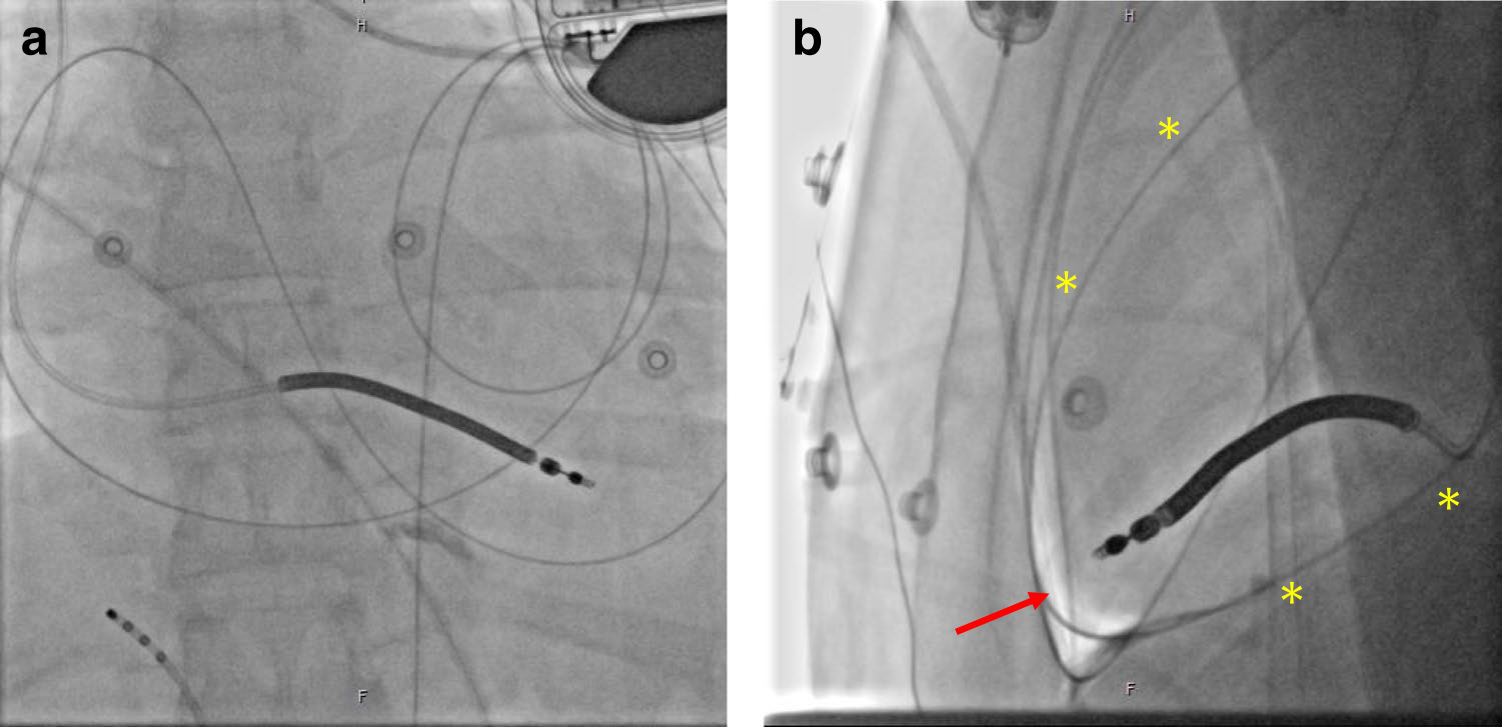
Anterior-oriented epicardial access. Correct anterior-oriented epicardial access in AP position (**A**) and LAO 90° (**B**). The wire courses around the heart (*yellow) indicating its position in the pericardial space around the right and left ventricles. Note also the small amount of air introduced by puncture (red →)

A total of 197 consecutive patients with structural heart disease referred for ablation of VT using an endo-epicardial or an epicardial-only (Fig. 1) approach between January 2016 and February 2022 were included in this study. This retrospective observational study is part of a large registry including all patients undergoing VT ablation at Heart Centre Bad Neustadt. All patients receiving at least one epicardial access for VT ablation were included. All patients had recurrent episodes of symptomatic sustained monomorphic VT documented by 12-lead ECG, Holter monitoring, or ICD interrogation.

All patients included in this study gave written informed consent to the respective procedures and the participation of the study. The study was approved by the institutional review board. The study was conducted according to the Declaration of Helsinki.

### Preprocedural workflow

In case of underlying atrial fibrillation (AF), a transesophageal echocardiography or a contrast-enhanced cardiac computer tomography (CT) was performed to rule out intracardiac thrombi prior to the VT procedure. According to our standard approach, a cardiac CT or late-gadolinium-enhancement magnetic resonance imaging (LGE-MRI) was acquired before the procedure if not contraindicated and processed to guide procedural planning and access sites based on location and transmural distribution of scar. Prior to epicardial puncture, oral anticoagulation with non-vitamin K oral antagonists (NOACs) was stopped 48 h before the procedure, vitamin K antagonists (VKAs) were stopped several days before to reach an INR level below 1.5. In these cases, bridging with low-molecular heparin (stopped the day of the procedure) or unfractionated heparin (syringe pump stopped 3 h before the procedure) was performed. All patients underwent VT ablation in the fasting state and under analgosedation using continuous propofol infusion in conjunction with morphine derivates. General anesthesia was only used when necessary due to hemodynamic instability or severely decompensated heart failure, when undergoing surgical subxiphoidal pericardial window or major impairment of respiratory function, at the discretion of the operator and only in the minority of patients.

Catheters were advanced in the right ventricle (transvenous approach) or left ventricle (retrograde access or transseptal approach) or both routes according to the presumed site of ablation target. Epicardial puncture was performed prior to heparinization. In a first step, a diagnostic catheter to the right ventricle was advanced; second, epicardial access was performed; and third, endocardial left ventricle access was performed with prior heparinization and endocardial mapping.

### Techniques of epicardial access and fluid management

The anterior-oriented approach was used in all procedures as institutional standard. A “dry” subxiphoid puncture under fluoroscopic guidance in AP and LAO 90° position was performed. After puncture of the skin, the Tuhoy needle was pushed forward under the xiphoid in a medial orientation towards the pericardium of the right ventricular free wall. In some cases, a contrast medium was injected to visualize “tenting” of the pericardium. Under fluoroscopical guidance, a dry puncture of the pericardial sack was performed resulting in a loss of resistance and accessibility of the pericardial space using a regular 35-in. wire. Adequate positioning of the wire in the pericardial space was verified by fluoroscopy documenting location of the wire around the heart silhouette in AP and 90° LAO projection. A 5F sheath was introduced over the wire and contrast medium was injected proving epicardial position of the sheath. A second wire (buddy wire) was inserted through the sheath which was then exchanged to a 9F steerable sheath (Agilis, Abott, St. Paul, USA) (Fig. 1).

If the anterior-oriented approach was not successful, an inferior-oriented puncture was performed as described previously by Sosa et al. [4]. In three cases, a surgical access to the epicardial space via a subxiphoid window was necessary because of failure to puncture using anterior and inferior approach. All epicardial accesses were supervised by a senior operator having performed over 500 epicardial punctures.

High-impedance irrigation fluid was introduced as standard for epicardial ablation in September 2019. As no half-normal saline (0.45%) is available in Germany dextrose 5% in half-normal saline (D5W) was used as standard irrigant for epicardial ablations [10, 11]. Before that, a standard irrigation fluid with normal saline (NS) was used. Irrigation rates were dependent on the ablation mode and the catheters used for ablation.

### Electrophysiological study

**Fig. 2:**
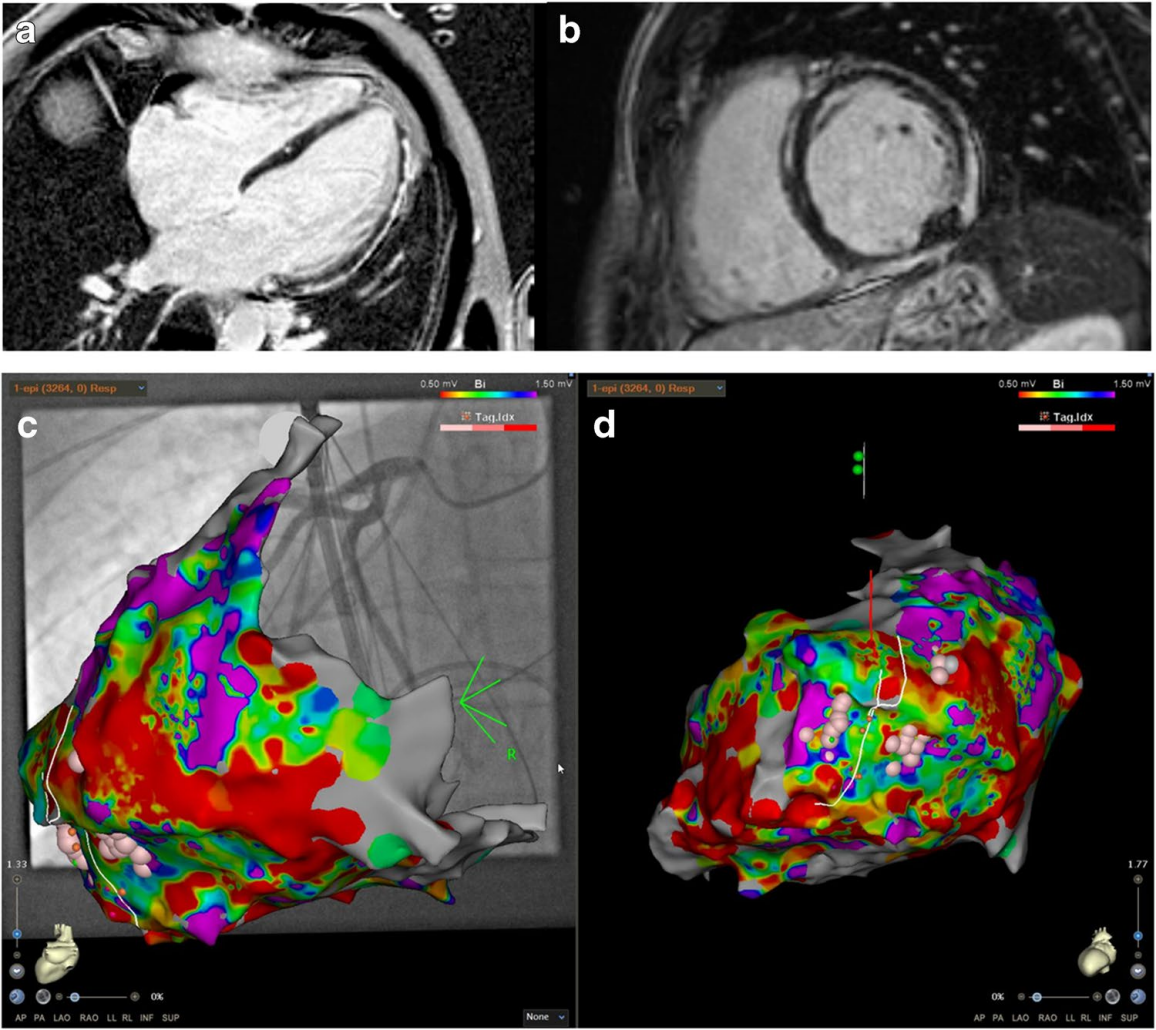
Preprocedural imaging using LGE-CMR to identify epicardial/midmyocardial substrate (A + B). Correspondingly, epicardial (C + D) map. Example of a patient undergoing preprocedural LGE-MRI imaging with evidence of epicardial substrate (A + B) with corresponding VT substrate on the epicardial mapping (C + D) reconstructed with CARTO-3 system (C: PA view showing patchy substrate epicardially, D: left lateral inferior view showing proposed VT channel based on preprocedural CT imaging and respective ablation lesions). The preprocedural MRI showed epicardial and partly intramural substrate inferior and inferolateral as well as inferoseptal

All epicardial procedures were performed using a three-dimensional electroanatomic mapping system (CARTO 2 or 3, BiosenseWebster, Diamond Bar, CA, USA; Ensite Precision, Abbott, St. Paul, MN, USA; Rhythmia, Boston Scientific, Natick, MA, USA) and performing electrical and structural substrate characterization using high-density multipolar mapping catheter (Pentaray, BiosenseWebster, Diamond Bar, CA, USA; Advisor HD Grid, Abbott, St. Paul, MN, USA; Intellamap Orion, Boston Scientific, Natick, MA, USA). Classical cutoff values for bipolar voltage (≤ 0.5 mV annotated as scar and of ≤ 1.5 mV but > 0.5 mV as low-voltage areas) [11] were used also for maps generated with multipolar high-density mapping catheters. Local abnormal ventricular potentials, late potentials, and fractionated low-amplitude potentials were additional criteria for identification of abnormal pathological ventricular tissue [12] (Fig. 2).

Before epicardial ablation coronary angiography was performed and no ablations were performed in close proximity to major epicardial vessels (< 1 cm). In all patients, the course of the phrenic nerve was documented using local pacing and no ablations were performed in sites with phrenic nerve capture. CT imaging–based localization of epicardial vessels and phrenic nerve was implied but not used as only identification of location in relation to ablation target.

All ablations were performed using irrigated contact force-sensing ablation catheters (ThermoCool SmartTouch SF, BiosenseWebster, Diamond Bar, CA, USA; or Tacticath, Abbott, St. Paul, MN, USA; or Intellanav MIFI XP ablation catheter, Natick, MA, USA). Endpoints of catheter ablation were effective ablation of late abnormal ventricular electrograms in areas with low bipolar voltage. Programmed ventricular stimulation at the end of the ablation procedure defined class of endpoint: elimination of the clinical VT (defined as partial success), elimination of any inducible VT defined as complete success. Ablation of all identified local abnormal activities and late potentials within a reasonable procedure duration was performed but not evaluated in all cases using remapping with high-density multielectrode mapping catheters.

### Post-ablation management

At the end of the procedure, all residual fluid was aspirated from the pericardial space. In all patients, 1000-mg methylprednisolone was administered to the epicardial space. If intraprocedurally no pericardial bleeding was documented, epicardial access was removed. If any sign of intraprocedureal pericardial bleeding was documented, a pigtail drain was inserted into the pericardial space and left in position until no further bleeding was documented. The epicardial pigtail was retracted after repeated exclusion of pericardial effusion using echocardiography and if maximal fluid extraction was below 50 ml/24 h. All patients underwent immediately after procedure ending, 6 h after the procedure, and the next day control echocardiography. Anticoagulation was resumed depending on the respective patients’ individual bleeding and thromboembolic risk and also at the discretion of the operator. In case of symptoms typical for pericarditis, medications with non-steroidal anti-inflammatory medication and colchicum were prescribed. All procedures were performed by highly experienced operators each having done > 50 endocardial VT ablation procedures before. All operators hold an invasive electrophysiology diploma of the European Heart Rhythm Association (EHRA) and the German Cardiac Society (DGK).

### Definition of complications and follow-up

Patient data were collected until discharge and all adverse events and deaths were documented. Procedural complications were recorded and classified in epicardial puncture-related and non-related. Major complications were defined as leading to interventional or surgical treatment, blood transfusion, or prolonged hospitalization. Other complications were considered minor.

Cardiovascular mortality was documented with the use of our electronic hospital information system. Furthermore, telephone interviews with patients or family members were performed at the end of the observational period to confirm the absence of primary and secondary endpoints.

Recurrence of clinical or any sustained ventricular arrhythmia (VA) was documented either by ICD interrogations during follow-up in most cases or by ECG. Clinical VT was considered if a VT with a CL equal or longer than the clinical VT was documented on ICD or having the same QRS morphology on event 12-lead ECG. Rehospitalization was verified according to telephone interviews and/or hospital reports. Lost to follow-up rate was low with 2%.

### Statistical methods

Quantitative data are presented as mean ± standard error of mean (SEM), median, and interquartile range (IQR), and ranges depending on the distribution of the data and were compared using Student’s *t* test for normally distributed data or the Mann–Whitney *U* test for nonparametric data. Deviations from a Gaussian distribution were tested by the Kolmogorov–Smirnov test. Spearman’s rank correlation for nonparametric data was used to test univariate correlations. Qualitative data are presented as absolute and relative frequencies and compared using the Chi^2^ test or Fisher’s exact test, as appropriate.

Continuous variables were evaluated by logistic regression; categorical variables were analyzed by contingency tables. Univariate regression analysis was performed for significant and clinically relevant variables. Multivariable regression analyses were used to determine risk factors for complications as well as predictors of VT recurrence and mortality in which all univariate significant and clinically relevant variables were included. The result of a statistical test was considered significant for *p* < 0.05; *p* values ≤ 0.1 were defined as a statistical trend. SAS, release 9.4 (SAS Institute Inc., Cary, NC, USA), and SPSS (Version 25, IBM Armonk, New York, USA) were used for statistics.

## Results

### Study population

A total of 197 patients (59.8 ± 15.3 years, 86% males) with a total of 239 epicardial procedures were included between 2016 and 2022. The most common underlying structural heart disease was dilated cardiomyopathy (38%), followed by ischemic cardiomyopathy (22%), myocarditis (19%), and ARVC (8%). Twelve patients (6%) had no documentable structural heart disease. Mean LVEF was 37 ± 14. Fourteen patients (7%) had prior open-heart surgery (six valve surgery, seven bypass surgery, one epimyocardial LV probe). Detailed baseline characteristics can be seen in Table 1.

**Table 1:** Baseline characteristics

Characteristic	All patients (*n* = 197)	
Age, median (range)	60 ± 15	
Males, *n* (%)	169	(86)
Cardiovascular risk factors, ***n*** (%)		
Arterial hypertension	137	(70)
Diabetes mellitus	43	(23)
Hyperlipidemia	109	(55)
Smoking	64	(33)
Cardiac family history	41	(21)
Comorbidities, ***n*** (%)		
CAD	59	(30)
Atrial fibrillation	89	(45)
Stroke	14	(7)
Chronic kidney disease	85	(43)
Liver cirrhosis	2	(1)
COPD	19	(10)
Asthma	4	(2)
Structural heart disease, ***n*** (%)		
Ischemic cardiomyopathy	43	(22)
Dilated cardiomyopathy	75	(38)
Myocarditis	38	(19)
Sarcoidosis	9	(5)
ARVC	15	(8)
HCM	3	(2)
HOCM	2	(1)
NCCM	1	(1)
Brugada	1	(1)
Idiopathic	12	(6)
Previous heart surgery, ***n*** (%)	14	(7)
Medication at admission, ***n*** (%)		
Beta-blocker	177	(90)
Amiodarone	93	(47)
OAK	92	(47)
DAPT	11	(6)
LVEF, %	37 ± 14	
Prior ICD, ***n*** (%)		
ICD	120	(61)
CRT-D	49	(25)
LifeVest	5	(3)
ICD indication, ***n*** (%)		
Primary prevention	51	(29)
Secondary prevention	125	(71)

*AAD*, antiarrhythmic drug; *ACHD*, adults with congenital heart defect; *ARVC*, arrhythmic right ventricular disease; *COPD*, chronic obstructive pulmonary disease; *CRT-D*, cardiac resynchronization therapy-defibrillator; *ES*, electrical storm; *HCM*, hypertrophic cardiomyopathy; *HOCM*, hypertrophic obstructive cardiomyopathy; *ICD*, implantable cardioverter defibrillator; *LVEF*, left ventricular ejection fraction

### Procedural characteristics

The median time between first VT and the epicardial ablation procedure was 30 days (from same day to 105 days post-index VT). For pre-procedural characterization of the ventricular substrate, planning access site (endocardial/epicardial, transseptal/retrograde) and guidance of intraprocedural substrate mapping cardiac CTs and LGE-MRIs were used in 55 procedures (23%) (39 procedures with MRI, 16 with CT) with significantly higher rates of preprocedural imaging among patients with D5W irrigation fluid (34% vs. 17%; 0.018). In total, 34% of all procedures were performed in patients with electrical storm. In 13 patients (5%), general anesthesia was used.

In 91% of procedures, the anterior-orientated epicardial access was successful (Fig. 1). Inferior access was achieved in 19 cases (12 cases with severe anterior adhesions; seven cases with difficult anatomy such as funnel chest) and in three patients epicardial access was gained surgically due to severe adhesions with epicardial puncture being unsuccessful. Epicardial access was possible in most patients, whereas in 14 cases access could not be achieved or achieved but no further mapping/ablation was possible due to severe adhesions and no further surgical access was tried. Pericardial adhesions were present in 38 patients (19%) (51 of procedures, 21%), in eight cases with prior myocarditis, 17 cases with prior epicardial access for VT ablation, nine cases with prior open-heart surgery, one patient with bronchial carcinoma due to radiation therapy, and one patient after chest trauma whereas in 14 patients no cause of adhesions was identified. In 21 patients epicardial adhesions limited epicardial mapping/ablation. In those 14 patients with prior thoracotomy an anterior access was successful in 11 (79%), whereas in two patients an inferior access was successful (14%) and one patient had surgical access (7%). However, nine had limiting pericardial adhesions, as mentioned above. In 20 patients (10%), epicardial mapping revealed no epicardial substrate and no epicardial ablation was performed. In addition, in two patients epicardial ablation targets were in close proximity of a major coronary artery and no ablation was performed. Thus, epicardial ablation was performed in 159 patients (81%) or in 196 cases (82%).

### Epicardial puncture-related complications and overall complications

A total of 40 complications occurred in the 239 epicardial procedures (17%). Eighteen (8%) of those were directly related to the epicardial puncture, mapping, or ablation including a total of four major epicardial puncture-related complications (1.7%) (one pericardial tamponade treated by pericardiocentesis, one pneumothorax needing pleural drainage, one puncture of the colon transversum with sheath insertion via the colon needing surgical repair, and one liver laceration needing emergency surgical repair).

The most common complication was pericardial bleeding with pericardial effusion > 5 mm but without hemodynamical impact and with conservative treatment in nine procedures (eight anterior, one posterior-oriented puncture; *p* = 0.731). The three patients with surgical access suffered from no complications.

Postinterventional pericarditis was diagnosed in 18 procedures (8%), also comparable distributed among groups. In two procedures minor groin complications occurred which could be treated conventionally. Post-ablation pneumonia occurred in four cases, all treated with antibiotics and healed without any sequelae. One patient (0.5%) suffered from a post-interventional stroke which occurred 16 h after the procedure. Two steam pops occurred. Four patients had deteriorated intraprocedural heart failure requiring catecholamine treatment (two of these patients died due to worsening intractable heart failure during hospitalization). A total of four patients (2.0%) died within 30 days including one due to intraprocedural pulseless electrical activity (0.5%), one patient due to electrical storm with concomitant cardiogenic shock 11 days after ablation (Table 2), and two with deteriorated heart failure (see above).

**Table 2:** Complications per procedure

Characteristic	Anterior access (*n* = 217)		Posterior access (*n* = 19)		Surgical access (*n* = 3)		All patients (*n* = 239)		*p* value
Epicardial puncture related complications, ***n*** (%)	13	(6)	5	(29)	0	(0)	18	(8)	**0.002**
Pericardial tamponade*	1	(1)	0	(0)	0	(0)	1	(0)	1.000
Pericardial effusion > 5 mm	8	(4)	1	(5)	0	(0)	9	(4)	0.731
Pneumothorax*	3	(1)	2	(11)	0	(0)	5	(2)	**0.008**
Abdominal organ puncture*	0	(0)	2	(11)	0	(0)	2	(1)	**0.006**
Pneumopericardium	1	(1)	0	(0)	0	(0)	1	(0)	1.000
Pericarditis	16	(7)	2	(11)	0	(0)	18	(8)	0.620
Complications not related to epicardial puncture, ***n*** (%)	18	(8)	4	(5)	0	(0)	22	(9)	0.113
Vascular access related	2	(1)	0	(0)	0	(0)	2	(1)	0.823
Hemodynamic instability	3	(1)	1	(6)	0	(0)	4	(2)	0.427
Pneumonia	3	(1)	1	(6)	0	(0)	4	(2)	0.427
AV conduction system-related	8	(4)	1	(6)	0	(0)	9	(4)	
Stroke	1	(1)	0	(0)	0	(0)	1	(0)	0.908
Steam pops	1	(1)	1	(6)	0	(0)	2	(1)	0.086
In-hospital mortality, ***n*** (%)	3	(1)	1	(6)	0	(0)	4	(2)	0.365

*AV*, atrio-ventricular

^*^Major complications: pericardial tamponade *n* = 1; abdominal organ puncture *n* = 2; pneumothorax *n* = 1

### Predictors of epicardial puncture-related complications

**Fig. 3:**
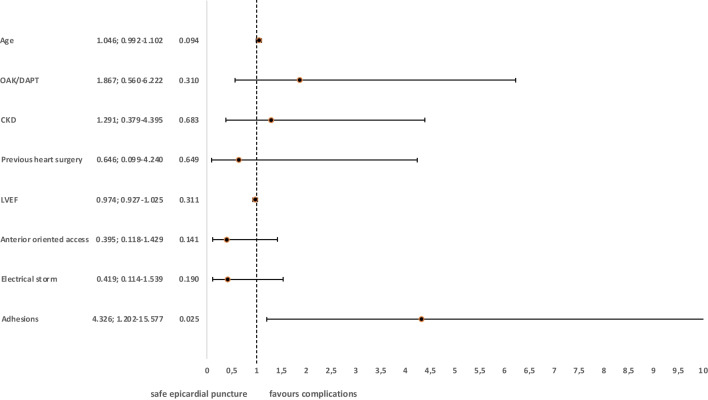
Predictors of complications related to epicardial access. CKD, chronic kidney disease; LVEF, left ventricular ejection fraction

In a multivariate logistic regression, only presence of pericardial adhesions (OR 4.326, 95% CI 1.202–15.577; *p* = 0.025) could be identified as strong negative prognostic factors in our cohort (Fig. 3).

To evaluate the possible influence of a learning curve, we divided the procedures into procedures between 2016 and 2018 and between 2019 and 2022. We found no statistically significant difference in terms of epicardial puncture-related complications (12/120 in the first half, 6/119 in the second half; *p* = 0.146).

### Different irrigation fluids

Patients ablated with NS were older, and had higher rates of chronic kidney disease and DCM with higher rates of CRT-D and primary preventive ICDs compared to D5W irrigation fluid. All other baseline characteristics were comparable (Table 3).

**Table 3:** Baseline characteristics in regard to irrigation fluids

Characteristic	NS (*n* = 93)		D5W (*n* = 66)		All patients (*n* = 159)		*p* value
Age, median (range)	62 ± 13		57 ± 16		60 ± 15		**0.029**
Males, *n* (%)	78	(84)	57	(86)	135	(85)	0.665
Cardiovascular risk factors, ***n*** (%)							
Arterial hypertension	66	(71)	41	(62)	107	(67)	0.241
Diabetes mellitus	20	(22)	13	(20)	33	(21)	0.782
Hyperlipidemia	54	(58)	28	(42)	82	(52)	0.052
Smoking	33	(36)	21	(32)	54	(34)	0.592
Cardiac family history	21	(23)	11	(17)	32	(20)	0.359
Comorbidities, ***n*** (%)							
CAD	26	(28)	17	(26)	43	(27)	0.758
Atrial fibrillation	45	(48)	26	(39)	71	(45)	0.261
Stroke	7	(8)	2	(3)	9	(6)	0.227
Chronic kidney disease	50	(54)	19	(29)	69	(43)	**0.002**
Liver cirrhosis	1	(1)	1	(2)	2	(1)	0.806
COPD	8	(9)	6	(9)	14	(9)	0.915
Asthma	3	(3)	0	(0)	3	(2)	0.141
Structural heart disease, ***n*** (%)							
Ischemic cardiomyopathy	21	(23)	13	(20)	34	(21)	0.662
Dilated cardiomyopathy	41	(44)	18	(27)	59	(37)	**0.031**
Myocarditis	15	(16)	18	(28)	33	(21)	0.078
Sarcoidosis	2	(2)	5	(8)	7	(4)	0.100
ARVC	7	(8)	6	(9)	13	(8)	0.723
HCM	2	(2)	1	(1)	3	(2)	0.772
HOCM	1	(1)	1	(1)	2	(1)	0.806
NCCM	0	(0)	1	(1)	1	(1)	0.411
Idiopathic	5	(5)	3	(5)	8	(5)	0.813
Previous heart surgery, ***n*** (%)	7	(8)	3	(5)	10	(6)	0.445
Medication at admission, ***n*** (%)							
Beta-blocker	85	(91)	156	(85)	141	(89)	0.199
Amiodarone	47	(51)	30	(46)	77	(48)	0.527
OAK	46	(49)	25	(38)	71	(45)	0.148
DAPT	5	(6)	2	(4)	7	(5)	0.627
LVEF, %	37 ± 13		39 ± 14		38 ± 14		0.272
Prior ICD, ***n*** (%)							
ICD	52	(56)	45	(68)	97	(61)	0.118
CRT-D	26	(28)	9	(14)	35	(22)	**0.032**
LifeVest	2	(1)	2	(3)	4	(3)	0.727
ICD indication, ***n*** (%)							
Primary prevention	30	(36)	11	(19)	41	(29)	**0.023**
Secondary prevention	53	(64)	48	(81)	101	(71)	

*ARVC*, arrhythmic right ventricular disease; *CAD*, coronary artery disease; *COPD*, chronic obstructive pulmonary disease; *CRT-D*, cardiac resynchronization therapy-defibrillator; *DAPT*, dual antiplatelet therapy; *HCCM*, non-compaction cardiomyopathy; *HCM*, hypertrophic cardiomyopathy; *HNS/D5W*, half-normal saline and non-ionic 5% dextrose in water; *HOCM*, hypertrophic obstructive cardiomyopathy; *ICD*, implantable cardioverter defibrillator; *LVEF*, left ventricular ejection fraction; *NS*, normal saline; *OAC*, oral anticoagulation

Ablation setup and access to the LV endo- and epicardially were comparable. Adhesions were more present among patients with NS irrigation (26% vs. 11%; *p* = 0.017).

In total, 17% of all patients were non-inducible before ablation and the mean number of inducible VTs was 1.9 ± 1.8 with higher numbers among patients with NS irrigation fluid (2.1 ± 1.9 vs. 1.6 ± 1.4; *p* = 0.049). Mean procedural duration and fluoroscopy times were higher among patients with NS, and ablation times comparable among groups.

Regarding the two different irrigation fluids, complication rates were comparable among all 196 patients who had successful epicardial access and were ablated in the epicardial space. Irrigation fluids had no impact on ablation-induced complications such as pericardial effusions (3 with D5W vs. 6 with NS; *p* = 0.662), pericardial tamponade (0 with D5W vs. 1 with NS; *p* = 1.000), steam pops (1 with D5W vs. 1 with NS; *p* = 0.813), and postinterventional pericarditis (7 with D5W vs. 11 with NS; *p* = 0.898).

Endpoints as ablation success were comparable between D5W and NS irrigation fluid management (Table 4). At the end of most procedures (89%), a programmed ventricular stimulation was performed. Acute partial procedural success was satisfying with non-inducibility of the clinical VT in 87% of all procedures (88% D5W vs. 86% NS; *p* = 0.812) and 74% of all patients were rendered non-inducible after the procedure (80% D5W vs. 71% NS; *p* = 0.161). In total, 6% of all patients still had inducible clinical VT at the end of the procedure (5% D5W vs. 7% NS; *p* = 0.611).

**Table 4:** Procedural data and intraprocedural success

Characteristic	NS (*n* = 93)		D5W (*n* = 66)		All patients (*n* = 159)		*p* value
Electrical storm	34	(37)	29	(44)	63	(40)	0.377
Elective procedure	38	(41)	25	(38)	63	(40)	0.705
General anesthesia	8	(9)	3	(5)	11	(7)	0.321
TSP	3	(3)	3	(5)	6	(4)	0.667
Anterior epicardial puncture	81	(87)	62	(94)	143	(90)	0.158
Inferior epicardial puncture	8	(9)	3	(5)	11	(7)	0.321
Surgical epicardial access	4	(4)	1	(1)	5	(3)	0.321
Adhesions	24	(26)	7	(11)	31	(20)	**0.017**
Non-inducible with VPS, n (%)	14	(15)	12	(19)	26	(17)	0.599
VTs inducible, *n*/patient	2.1 ± 1.9		1.6 ± 1.4		1.9 ± 1.8		**0.049**
Clinical VT CL, ms	343 ± 85		364 ± 78		356 ± 85		0.177
Procedural duration, min	208 ± 66		182 ± 45		197 ± 59		**0.006**
Fluoroscopy duration, min	25.6 ± 13.2		18.7 ± 10.3		22.6 ± 12.6		**0.001**
Ablation time, min	32.1 ± 22.2		32.7 ± 19.8		32.3 ± 21.1		0.870
Betablocker at discharge, *n* (%)	90	(99)	60	(91)	150	(96)	**0.017**
Amiodaron at discharge, *n* (%)	37	(41)	24	(36)	61	(39)	0.586

*CL*, cycle length; *HNS/D5W*, half-normal saline and non-ionic 5% dextrose in water; *NS*, normal saline; *TSP*, transseptal puncture; *VPS*, ventricular programmed stimulation; *VT*, ventricular tachycardia

### Hospital course

The mean hospitalization time was 15.0 ± 14.9 days in our series. In total, 183 patients were transferred to intensive care medicine (= specialized VT unit) after the procedure and the mean time spend on intensive care unit was 7.4 ± 13.7 days. A total of 38 patients suffered from intrahospital VT recurrence (25 with NS, 13 with D5W; *p* = 0.488) and 27 of them underwent a second VT ablation procedures during index hospitalization after a mean of 9.8 ± 9.4 days. The 30-day mortality was 2.0% (see above).

### VT recurrences and follow-up

**Fig. 4:**
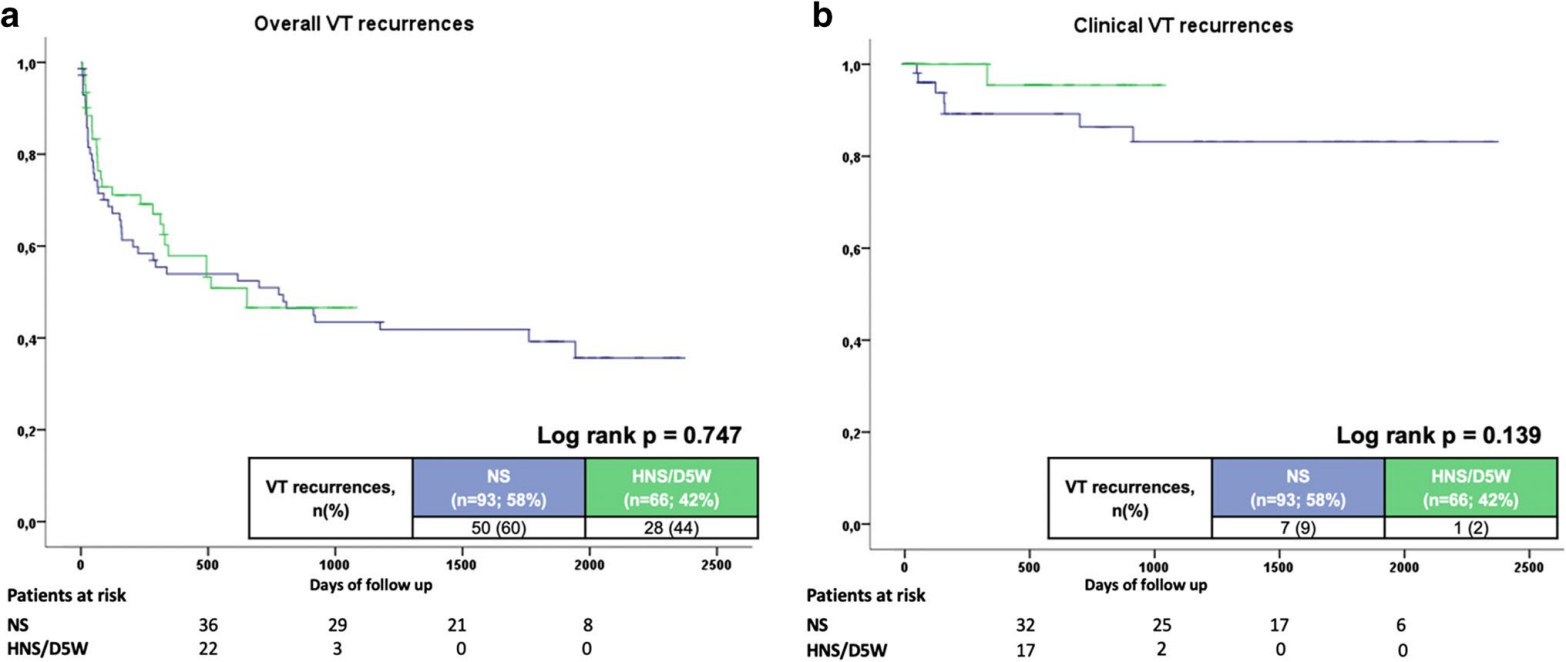
Kaplan–Meier curves illustrating recurrences rates among different irrigation fluids: (A) overall VT recurrences, (B) clinical VT recurrences

The median follow-up time was 37 ± 23 months. New VTs were documented in 53% of all patients but only 8% of patients had recurrence of their clinical VT. It should be emphasized that a steep slope at the beginning of follow-up can be seen representing the high recurrence rates early after VT ablation. Patients with D5W showed comparable overall recurrence rates (60% vs. 44%; log-rank *p* = 0.747) and clinical VT recurrences (9% vs. 2%; log-rank *p* = 0.139) (Table 5; Fig. 4). Using multivariable regression analysis, electrical storm (HR 1.903, 95% CI 1.043–3.472; *p* = 0.036) and epicardial adhesions (HR 2.174, 95% CI 1.046–4.516; *p* = 0.037) were identified as independent predictors of VT recurrences, whereas amiodarone treatment was protective (HR 0.435, 95% CI 0.215–0.879; *p* = 0.021). Notably, irrigation fluid management (HR 1.028, 95% CI 0.642–1.644; *p* = 0.909) and preprocedural imaging (HR 0.734, 95% CI 0.486–1.110; *p* = 0.142) did not reveal a statistically significant impact on VT recurrences (Table 6).

**Table 5:** Primary and secondary endpoints

Characteristic	NS (*n* = 93)		D5W (n = 66)		All patients (*n* = 159)		*p* value
**Primary endpoint, *n* (%)**							
Any VT recurrence	50	(60)	28	(44)	78	(53)	**0.047**
Clinical VT recurrence	7	(9)	1	(2)	8	(6)	0.073
**Secondary endpoints,* n* (%)**							
Heart failure–related rehospitalization	14	(15)	10	(15)	24	(12)	0.986
LVAD/HTX	6	(6)	2	(3)	8	(4)	0.331
Cardiovascular mortality	25	(29)	3	(5)	28	(18)	**0.001**

*HTX*, heart transplantation; *HNS/D5W*, half-normal saline and non-ionic 5% dextrose in water; *LVAD*, left ventricular assist device; NS, normal saline; *VT*, ventricular tachycardia

**Table 6:** Uni- and multivariable Cox regression for VT recurrence

Variable	Univariable			Multivariable		
	HR	95% CI	*p* value	HR	95% CI	*p* value
Ablation time	1.008	0.996 – 1.020	0.193	-	-	-
Adhesions	1.800	1.582 – 3.003	0.086	2.174	1.046 – 4.516	**0.037**
Elective procedure	0.818	0.517 – 1.295	0.392	-	-	-
Full ablation success	0.770	0.526—1.128	0.179	-	-	-
Hemodynamically relevant VT	1.207	0.739 – 1.973	0.452	-	-	-
Electrical storm	1.834	1.242 – 2.425	**0.021**	1.903	1.043 – 3.472	**0.036**
Number of VT inducible	1.057	0.934 – 1.195	0.379	-	-	-
LVEF	0.998	0.981 – 1.016	0.851	-	-	-
Amiodarone at discharge	0.608	0.361 – 1.022	0.060	0.435	0.215 – 0.879	**0.021**
CL clinical VT	0.999	0.996 – 1.002	0.453	-	-	-
Preprocedural imaging	0.797	0.510 – 1.247	0.320	-	-	-
DW5 irrigation fluid	1.028	0.642 – 1.644	0.909	-	-	-

*CI*, confidence interval; *CL*, cycle length; *HNS/D5W*, half-normal saline and non-ionic 5% dextrose in water; *HR*, hazard ratio; *LVEF*, left ventricular ejection fraction; *VT*, ventricular tachycardia

In total, 24 patients (12%) were rehospitalized due to acute or worsening heart failure. Eight patients (4%) received a permanent left ventricular assist device or heart transplantation during follow-up.

The overall long-term cardiovascular mortality rate was high with 18% during follow-up. Cardiogenic shock due to terminal heart failure was the most common cause of death in our cohort (16 patients (10%)). Five patients died due to recurrent incessant VTs (3%), two due to sepsis (1%), and one patient died after heart surgery. In four patients, the cause of death remained unclear (all with severely reduced LV function).

**Fig. 5:**
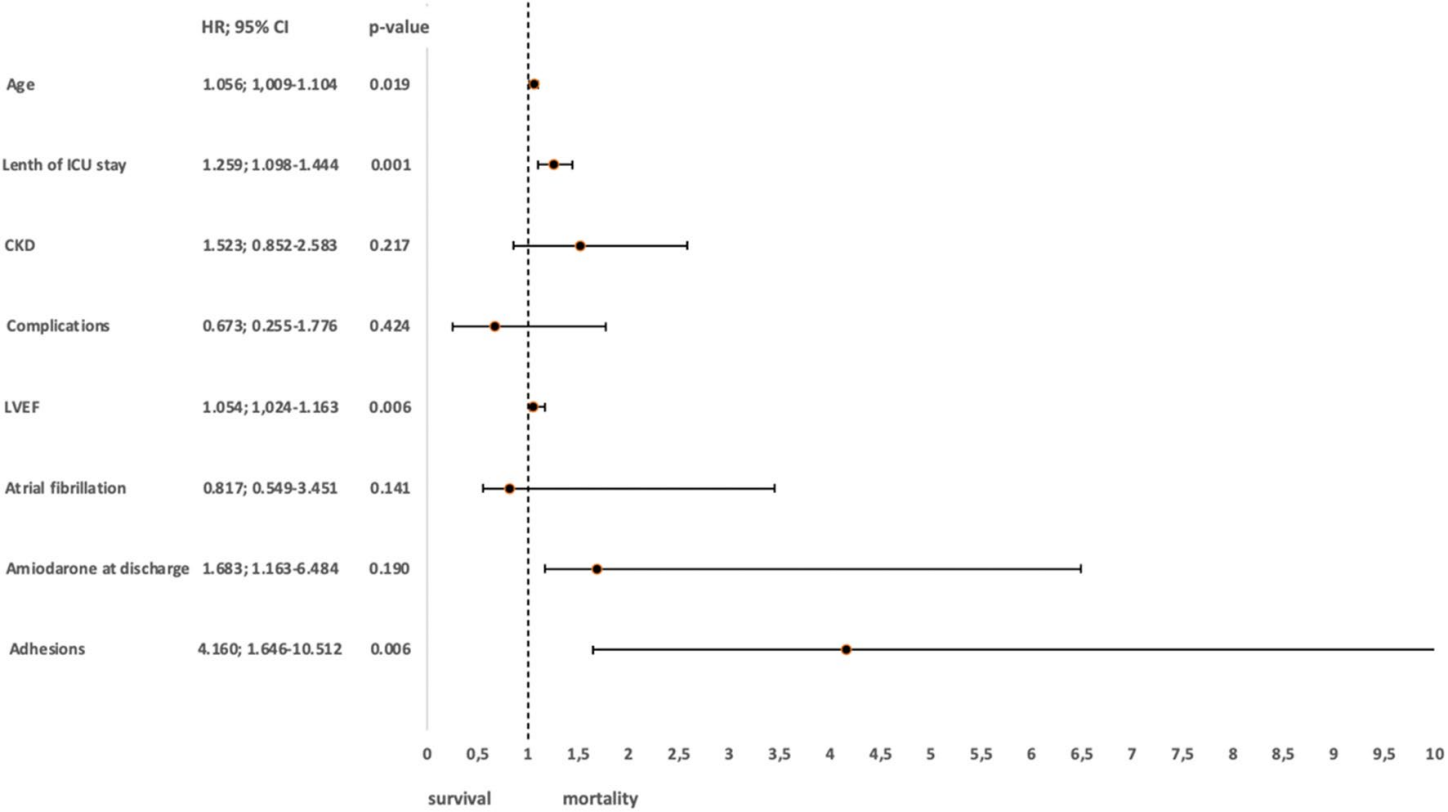
Predictors of cardiovascular mortality during follow-up. CKD, chronic kidney disease; LVEF, left ventricular ejection fraction

Using multivariable Cox regression models, several factors for cardiovascular mortality could be identified: increasing age (HR 1.056, 95% CI 1.009–1.104; *p* = 0.019), presence of adhesions (HR 4.160, 95% CI 1.646–10.512; *p* = 0.003), prolonged ICU hospitalization time (HR 1.259, 95% CI 1.098–1.444; *p* = 0.001), and decreased LVEF (HR 1.054, 95% CI 1.024–1.163; *p* = 0.006) (Fig. 5).

## Discussion

This study reports the experience of a high-volume EP center about the safety of epicardial access for VT ablation using mainly an anterior-oriented approach and the safety and efficacy of two different irrigation fluids. The major findings of this study are (1) the epicardial puncture is safe with major complications in 1.7% and overall complication rate of 8%; (2) using an epicardial access resulted in high acute ablation success with complete elimination of the clinical VT; (3) D5W irrigation fluid has a comparable efficacy and safety profile compared to NS; (4) during follow-up cardiovascular mortality rates were high with 18% and (5) independent predictors of mortality were increasing age, epicardial adhesions, low LVEF, and length of ICU stay.

### Incidences of complications related to epicardial puncture

Since the first report of successful percutaneous epicardial VT ablation in the late 1990 by Sosa et al. in three patients with Chagas disease, the number of epicardial ablation procedure increased rapidly. The inferior-oriented approach is the most commonly used access route but large cohorts including 200 consecutive patients using the inferior approach reported relevant complications in 7% [13]. Other studies also reported a high risk for access-related complications with pericardial tamponade as most frequent complication in more than 5% up to even 30% [6, 14, 15]. Puncture of abdominal organs has been reported as a specificity of the inferior approach as anatomically parts of the intraperitoneal space have to be crossed. In our series an anterior approach has been used as standard for epicardial access and only if not effective the inferior-oriented approach was performed in 19 procedures (8%). Complications were significantly more common when using the inferior approach (almost 30% of cases) in our experience, including two major complications needing emergent abdominal surgery for liver laceration and colon puncture. It is important to note that no patient required emergency cardiac surgery, but the only surgical interventions needed were abdominal. Abdominal complications were also not noted with an anterior approach in another large single-center experience [16]. “Easier” pericardial access with the anterior access might be due to mostly anterior-located pericardial fluid distribution in supine position as shown in CT imaging studies [17]. Another more recent study could confirm the reasonable safety profile of an anterior-oriented approach for VT ablation, in which no bleeding complication and need for emergency cardiac surgery was reported [18]. However, major differences in the patients’ characteristics must be mentioned; patients in the abovementioned study were significantly younger and revealed less comorbidities, and only few patients were on anticoagulants. Furthermore, a significant part suffered from ARVC as underlying heart disease and therefore no LV mapping was performed and no periinterventional heparin was administered further reducing bleeding risk. In our cohort pericardial bleedings using the anterior approach were present in only eight of 217 procedures (4%). The complication rate documented in our cohort appears lower compared to other series [16, 19]. In summary, using the anterior access route for epicardial VT ablation appears to be safe in experienced hands and allows access to all areas of VT targets. Using the inferior approach only as primary access in patients after prior cardiac surgery due to the location of adhesions anteriorly has become our standard. Independent to the access approach epicardial VT ablation was effective and eliminated the clinical VT in over 85% of cases.

### Influence of HNS or D5W irrigation fluid

RF energy delivery is affected by irrigation type and the irrigation cloud may lead to shunting of RF energy if a milieu with lower impedance or high electrical conductivity is created. Therefore, changing the irrigation fluid to half normal saline (0.45%) produces higher lesion volumes but also higher occurrence of steam pops [20]. Using higher impedance irrigants in the pericardial space may help to direct energy delivery to the myocardium and prevent untoward energy shunting to the irrigation fluid [10]. We have used a low-impedance irrigation fluid as standard in our cases and have not documented higher incidences of complications including steam pops or damage to epicardial structures such as coronary arteries or phrenic nerve. Furthermore, acute ablation success with non-inducibility of the clinical VT was high, as 88% and 80% of all patients were rendered non-inducible for any VT after ablation using D5% as epicardial standard irrigation fluid. Our data strengthen the current knowledge that low-ionic irrigation fluid such as D5W is safe with high acute success rates for epicardial VT ablation. In addition, long-term recurrences rates did not differ significantly between irrigation fluids.

### Complication prevention

Although overall occurrence of pericardial tamponade was very low in our experience, some general considerations can help preventing this complication. An important issue is the risk for accidental RV puncture, which does not necessarily lead to severe bleeding if the sheath is not advanced into the RV. To minimize this risk several different techniques and tools have been published [14]. A LAO 90° guided technique with a micropuncture needle [7, 21] or prior insufflation of CO2 as previously described to prevent unintentional RV puncture may be options [22].

### Clinical implications

Our manuscript reports a single-center experience of a high-volume EP center for VT ablations using routinely an anterior oriented and only as “backup” technique an inferior-oriented approach for epicardial access. These results encourage the high safety rates of epicardial ablations in experienced EP centers. Epicardial mapping and ablation may be performed early in experienced centers if epicardial substrate is assumed or documented in preprocedural imaging. In our series, we did not identify complications needing cardio-surgical backup but abdominal complications using the inferior approach were documented needing surgical emergency repair [23].

Routine use of D5W as irrigation fluid for epicardial RF ablation was shown to have a comparable safety profile as standard 0.9% saline irrigation and created comparable efficacy and high acute ablation success rates. Further analysis should emphasize the importance of preprocedural imaging and may focus on patients with “hard-to-reach” midmyocardial substrates.

## Study limitations

This study is a single-center, non-randomized study representing the experience of a high-volume EP center performing a high number of epicardial EP procedures annually. Low complication rates may be related to the high experience of the individual operators and may not apply to other settings. Inter-operator change was low and a learning curve of the operators cannot be excluded. The study represents the outcomes after a single VT ablation procedure and repeated VT ablation could change long-term outcomes. The study represents a patient cohort with advanced heart failure and severely reduced left ventricular function. Patients with an earlier disease state may be different from those included in this study. Prior open-heart surgery is considered as a relative contraindication for interventional epicardial approach. However, as shown in this study, it can be a safe bailout approach in experienced centers, if prior endocardial ablations failed and epicardial focus is suspected. Notably, all of those patients in this study had at least one prior unsuccessful endocardial ablation. This was a retrospective analysis.

## Conclusions

In this large single-center experience, epicardial ablation of VTs was safe with a low risk of major complications, especially if no adhesions were present and an anterior access route was used. Despite high acute ablation success leading to non-inducibility of all VTs in almost 80% of patients, long-term recurrences were documented in a substantial proportion of patients. In this cohort of severely sick patients epicardial VT ablation is associated with a low 30-day mortality of 2% but long-term cardiovascular mortality mostly due to worsening heart failure is high (18%). The routine usage of D5W irrigation fluid is safe and delivers comparable acute and long-term results compared to normal saline irrigation.

## Data Availability

Data is available from the corresponding author upon reasonable request.
